# PMN-MDSC are a new target to rescue graft-versus-leukemia activity of NK cells in haplo-HSC transplantation

**DOI:** 10.1038/s41375-019-0585-7

**Published:** 2019-10-04

**Authors:** Nicola Tumino, Francesca Besi, Anna Laura Di Pace, Francesca Romana Mariotti, Pietro Merli, Giuseppina Li Pira, Federica Galaverna, Angela Pitisci, Tiziano Ingegnere, Andrea Pelosi, Linda Quatrini, Enrico Munari, Franco Locatelli, Lorenzo Moretta, Paola Vacca

**Affiliations:** 1 Immunology Research Area, IRCCS Bambino Gesù Pediatric Hospital, Rome, Italy; 2 Department of Pediatric Hematology/Oncology, IRCCS Bambino Gesù Children’s Hospital, Rome, Italy; 3 Department of Pathology, Sacro Cuore Don Calabria, Negrar, VR Italy; 4 Department of Diagnostics and Public Health, University of Verona, Verona, Italy; 5 Department of Pediatrics, Sapienza, University of Rome, Rome, Italy

**Keywords:** Transplant immunology, Innate immunity

## To the Editor:

αβT cell- and B cell-depleted HLA-haploidentical haematopoietic stem cell transplantation (haplo-HSCT) is a life-saving therapeutic option to treat patients with high-risk leukemia lacking an HLA-compatible donor [1]. In addition to hematopoietic stem cells (HSC), this manipulated graft contains mature donor-derived NK and γδT cells, both exerting graft-versus-leukemia (GvL) activity and control of infections at early stages after transplantation [2]. Despite a satisfactory clinical outcome both in acute lymphoid and myeloid leukemia patients (~70% probability of survival at 5 years), differently from the HSCT using “pure” CD34^+^ precursors, the contribution of alloreactive NK cells (displaying KIR/HLA-I mismatch in the donor versus recipient direction), was found to be marginal [3, 4]. Notably, the G-CSF-induced mobilization of HSC in the donor causes relevant increases of different myeloid cells [5]. About 10% of donors, being “poor mobilizers”, were further treated with plerixafor. However, no substantial differences were detected in cellular composition of the graft (data not shown). Since myeloid-derived suppressor cells (MDSC) could exert an inhibitory effect on NK- and γδT-cell effector function [6–9], it is crucial to assess whether such cells are present in the graft, possibly interfering with GvL activity. Human MDSC are classified in two major subsets: monocytic (Mo) (CD45^+^Lin^-^HLA-DR^−/low^CD33^+^CD11b^+^CD14^+^CD66b^−^) and polymorphonuclear (PMN) (CD45^+^Lin^−^HLA-DR^−/low^CD33^+^CD11b^+^CD14^−^CD66b^+^), identified based on different surface antigens [6]. The expansion of these cell subsets was observed during pathological conditions, characterized by an high status of inflammation, such as viral/bacterial infections, autoimmune diseases, and tumors [10].

**Fig. 1:**
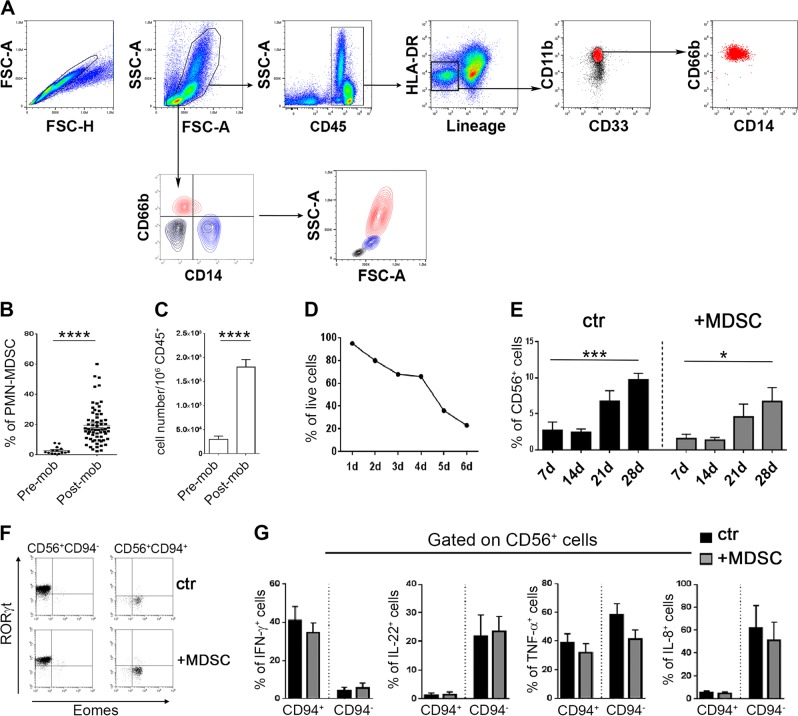
Presence of PMN-MDSC in the apheresis of G-CSF-mobilized donors and their effect on NK/ILC differentiation from HSC. **a–c** Mononuclear cells present in the apheresis were analyzed by flow-cytometry for the expression of specific markers allowing the identification of MDSC subsets. **a** Gating strategy adopted in one representative experiment out of 70 performed. **b** Percentages and **c** absolute numbers of PMN-MDSC (CD66b^+^ cells) in different donors before (pre) and after (post) mobilization (mob). **d** Percentages of PMN-MDSC viable cells at different time points. **e–g** In these experiments, CD34^+^ HSC were isolated from the apheresis of G-CSF-mobilized donors and cultured with cytokine-medium either in the absence (ctr) or in the presence (+MDSC) of PMN-MDSC (at 1/1 ratio) and analyzed by flow-cytometry for: **e** percentages ± SEM of CD56^+^ cells at different time points (*n* = 7); **f** RORγt and Eomes transcription factor expression in gated CD56^+^CD94^+^ cells (NK cells) and CD56^+^CD94^−^ (ILC3) at day 40 of culture. One representative experiment out of seven performed; **g** expression of informative intracellular cytokines (IFN-γ, IL-22, TNF-α, and IL-8) at day 40 in CD56^+^CD94^+^ or CD56^+^CD94^−^ cells after stimulation with PMA+Ionomycine+IL-23. Percentage ± SEM (*n* = 3–7). A *p* value ≤ 0.05 was considered statistically significant. *p ≤ 0 .05; **p ≤ 0 .01; *** *p* ≤ 0.001; **** *p* ≤ 0.0001; ns = not significant. Where not indicated, the data were not statistically significant

In this study, we show that PMN-MDSC are present in high proportions in the graft of αβT cell- and B cell-depleted transplants and exert a sharp inhibition on the effector functions of mature NK cells co-infused with the graft. A first set of experiments by flow-cytometry revealed that PMN-MDSC, but not Mo-MDSC, were highly enriched in mononuclear cell populations isolated from peripheral blood (PB) of G-CSF-mobilized haploidentical donors (Fig. 1a–c, Supplementary Materials and methods). Importantly, PMN-MDSC were further enriched in the αβT- and B-cell-depleted grafts (in which they were >10-fold more than NK cells, data not shown). Of the 70 donors analyzed, 47% were male, median age = 40 years and 53% female, median age = 37 years (see Supplemental Materials and methods). Since mature PMN cells typically display a short life span (<24 h) [11], we analyzed the in vitro survival of PMN-MDSC isolated from mobilized donors. The percentage of viable cells decreased very slowly from day 1 to day 4 of culture, while it dropped sharply only after day 5 (Fig. 1d). These results indicate that donor-derived PMN-MDSC are characterized by a relatively prolonged life span, possibly reflecting their immature stage and/or the treatment with G-CSF. Given the high proportion of PMN-MDSC in the αβT cell- and B cell-depleted graft, first we asked whether they could compromise the differentiation of HSC towards NK cells. Figure 1e shows only a partial inhibitory effect on HSC differentiation towards CD56^+^ cells. We further analyzed the composition and the functional capabilities of CD56^+^ progenies obtained in the presence of PMN-MDSC. As shown in Fig. 1f, both the EOMES-expressing CD56^+^CD94^+^, possibly corresponding to maturing NK cells, and the RORγt-expressing CD56^+^CD94^−^, possibly corresponding to ILC3, were present. The two subsets could be unequivocally attributed to NK and ILC3, respectively, as revealed by the analysis of the expression of informative cytokines. Thus, CD94^+^ cells differentiated from both control and MDSC-containing cultures produced IFN-γ and TNF-α, but not IL-22 and IL-8. On the other hand, CD94^−^ produced both IL-22 and IL-8, as well as TNF-α, but not IFN-γ (Fig. 1g). These data indicate that the CD94^+^ and CD94^−^ cell subsets are both functional and their sets of cytokines indeed correspond to those typical of NK cells and ILC3, respectively. Taken together, these experiments indicate that PMN-MDSC could exert some inhibitory effect. However, the respective progenies display both phenotypic and functional characteristics of the two mature subsets.

**Fig. 2:**
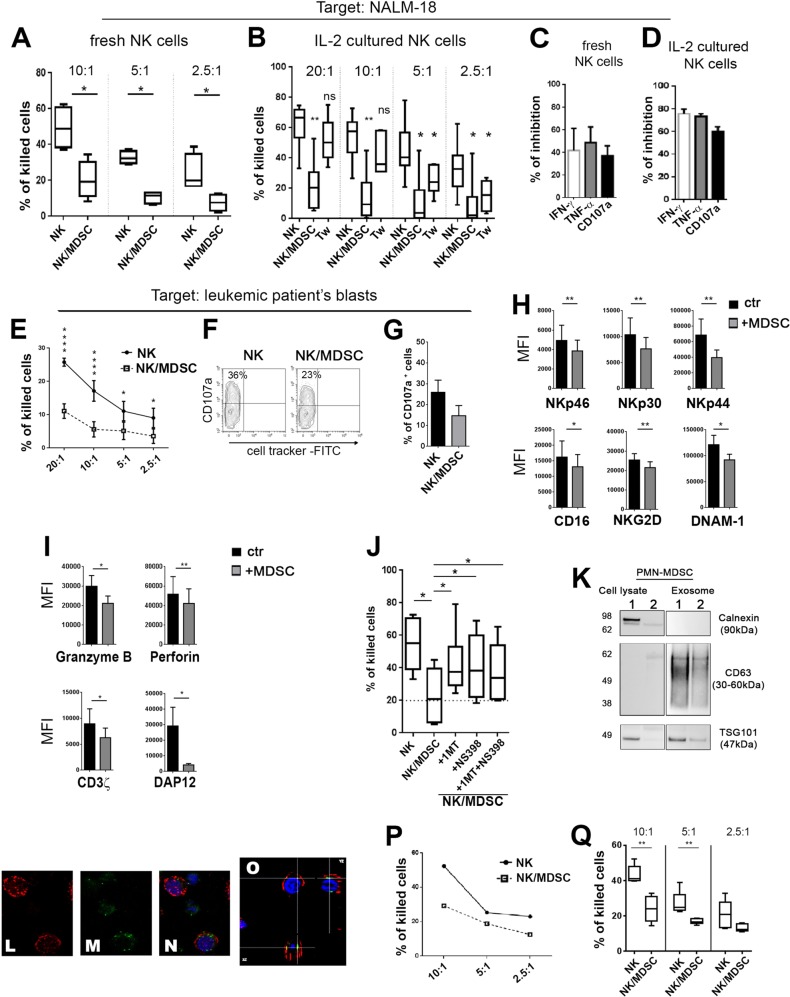
PMN-MDSC inhibit NK-cell function via IDO, PGE2, and exosomes. **a**–**d** In these experiments, the acute lymphoblastic leukemia cell line NALM-18 was used as target to asses either the NK-cell cytolytic activity or their cytokine production. Freshly isolated or IL-2-expanded NK cells were cultured either alone (NK) or in the presence of PMN-MDSC at 1/1 ratio. After 48 h of co-culture, PMN-MDSC were removed by magnetic cell separation. The resulting NK cells were used as effector cells (indicated as NK/MDSC) in the cytolytic, cytokine and degranulation assays. Percentages of killed target cells by **a** freshly isolated (*n* = 4) or **b** IL-2-cultured NK cells (*n* = 6). **b** NK cells were cultured either alone or under cell-to-cell contact (NK/MDSC) or under transwell (Tw) conditions as indicated in the figure. Cytokine production and degranulation of **c** freshly isolated (*n* = 3) or **d** IL-2 expanded NK cells (*n* = 5). Bars indicate the PMN-MDSC-mediated inhibition (% ± SEM), referred as the ratio between NK/MDSC co-cultures and NK cells cultured alone (arbitrarily normalized to 100). **e**–**g** Evaluation of the effect of donor PMN-MDSC on autologous NK-cell cytotoxicity and degranulation capability (CD107a) against recipient leukemia blasts. **e** Percentages of killed patient’s blasts by freshly isolated donor NK cells (*n* = 5). **f** One representative contour plots and **g** percentages ± SEM of CD107a expression against patient’s leukemia blasts (*n* = 3). In **h**–**i** were analyzed the mechanism(s) involved in the NK-cell inhibition. IL-2-expanded NK cells were cultured either alone (ctr., black bars) or in the presence of PMN-MDSC at 1/1 ratio. After 48 h of co-culture, PMN-MDSC were removed by magnetic cell separation. The resulting NK cells (referred as +MDSC, gray bars) were analyzed. Mean fluorescence intensity (MFI) ± SEM of the indicated (**h**) surface markers (*n* = 12), (**i**) granzyme B (*n* = 8), perforin (*n* = 9) and signaling molecules CD3ζ (*n* = 9), and DAP12 (*n* = 6). **j** IL-2-expanded NK cells were cultured either alone or with PMN-MDSC (at 1/1 ratio) in the absence or in the presence of 1-MT (IDO inhibitor) and/or NS398 (PGE2 inhibitor). The cytolytic activity of NK cells cultured under different conditions was tested against NALM-18 cells (*n* = 6). Percentages ± SEM of killed cells (E/T ratio used was 10/1). **k** Western blot analysis of calnexin, CD63, and TSG101 in PMN-MDSC cell lysates and in PMN-MDSC-derived exosome. **l**–**o** Confocal microscopy of NK cells incubated for 48 h with exosomes purified from PMN-MDSC (5 µg/5 × 10^5^ cells). Confocal microscopic analysis of l CD56^+^ NK cells (red), **m** PKH67^+^-exosomes (green), **n** merge of CD56^+^ and exosome, nuclei in DAPI (blue); **o** confocal z-stack of merged section. Magnification ×60. **o**, **p** Percentages of killed NALM-18 cells after incubation with PMN-MDSC-derived exosomes. **p** One representative experiment out of six performed and statistical analysis. A *p* value ≤ 0.05 was considered statistically significant. *p ≤ 0 .05; **p ≤ 0 .01; ***p ≤ 0.001; ****p ≤ 0.0001; ns = not significant. Where not indicated, the data were not statistically significant

An important improvement achieved with the αβT cell- and B cell-depleted haplo-HSCT, over the “pure” CD34^+^ HSC, in the clinical outcome of high-risk leukemia patients is related to the co-infusion of mature donor NK and γδT cells [3]. Accordingly, another central question was whether PMN-MDSC, co-infused in the graft with NK and γδT cells, were potentially capable of compromising the function and antileukemia activity of these effector cells. To this end, PMN-MDSC, freshly purified from apheresis of mobilized donors, were cultured for 48 h with allogenic, freshly isolated or IL-2-cultured NK cells at 1/1 ratio. Figure 2a, b shows a sharp inhibitory effect on the cytolytic activity (expressed as % of killed target cells) of both fresh and IL-2-cultured NK cells against the NALM-18 leukemia cell line. Similarly, the cytokine production in both freshly isolated and IL-2-cultured NK cells co-cultured with PMN-MDSC was strongly inhibited as compared with NK cells cultured alone (Fig. 2c, d). In addition, the expression of CD107a, a marker of cytolytic cell degranulation (and of cytolytic activity) was impaired (Fig. 2c, d). Similar results were obtained using as target cell the K562 erythroleukemia cell line (data not shown). In order to obtain a direct information on the effect of PMN-MDSC on donor NK-cell-mediated killing of patient’s leukemia blasts, experiments were performed using these blasts as target cells in a cytolytic assay. Cytolytic activity of donor NK cells co-cultured with PMN-MDSC was strongly inhibited (Fig. 2e). The PMN-MDSC inhibitory effect was confirmed also by the analysis of CD107a expression in donor NK cells upon interaction with leukemia blasts (Fig. 2f, g). These data further support the notion that, indeed, PMN-MDSC contained in the graft may exert an early inhibitory effect on NK cell-mediated GvL activity.

Since an inhibitory effect was detected also under transwell conditions (Fig. 2b), these data suggested the involvement of PMN-MDSC-derived soluble factors. Previous studies indicated that different inhibitory factors/cytokines may affect the surface expression or the signaling capability of major activating NK receptors involved in tumor cell killing [12]. As shown in Fig. 2h, i, the expression of NKp46, NKp30, NKp44, CD16, NKG2D, DNAM-1, Granzyme B, and Perforin was significantly reduced when NK cells were co-cultured with PMN-MDSC. In addition, the expression of the ITAM-bearing adapter polypeptides CD3ζ and KARAP/DAP-12 mediating signal transduction of these activating receptors, was inhibited (Fig. 2i). Notably, the use of the IDO inhibitor 1MT and/or NS398 (PGE-2 inhibitor) could partially restore the NK cytolytic activity, thus indicating that IDO and PGE2 are involved in the suppression (Fig. 2j). Supplementary Fig. 1a, b shows the presence of IDO1 and IDO2 proteins and mRNA in PMN-MDSC. In contrast, neither nor-NOHA (l-arginase inhibitor) nor anti-TGF-β mAbs had any effect (data not shown).

Different physiologic and pathologic processes are induced/modulated by exosomes released from different cell types [13]. Thus, we assessed whether also PMN-MDSC-derived exosomes contributed to the inhibitory effect on NK cell function. Exosomes were isolated from PMN-MDSC and the presence of exosome markers (CD63 and TSG101) documented by western blot (Fig. 2k). In contrast, the endoplasmic reticulum protein calnexin was present only in PMN-MDSC lysates, indicating that our purified preparations of exosomes were not contaminated by cellular components (Fig. 2k). Moreover, PMN-MDSC-derived exosomes were internalized and clearly detectable in the cytoplasm of NK cells (Fig. 2l–o). To assess their immunomodulatory potential, NK cells were cultured in the presence of PMN-MDSC-derived exosome. A significant impairment of NK-cell cytolytic activity was detected (Fig. 2p, q).

Our study indicates that PMN-MDSC derived from G-CSF-mobilized donors can exert a potent inhibitory effect on the antileukemia activity of donor mature NK cells in peripheral blood stem cell transplant recipients. The inhibitory effect involves different mechanisms, including production of soluble factors and exosomes. Notably, in the αβT cell- and B cell-depleted haplo-HSCT setting, the contribution of NK cell alloreactivity to prevent leukemia relapses was marginal, possibly reflecting a substantial impairment of the whole NK cell function [3]. No information exists on the degree of in vivo co-localization of grafted PMN-MDSC and NK cells. Of note, however, a strong inhibitory effect in vitro occurred also at 1/1 ratio, while PMN-MDSC largely outnumber (>10-fold) NK cells in the graft, thus rendering likely the occurrence of in vivo interactions and consequently of an inhibitory effect. Unlike NK cells, γδT lymphocytes were mostly resistant to the inhibitory effect of PMN-MDSC (data not shown). Notably, patients given αβT cell- and B cell-depleted haplo-HSCT frequently received zoledronic acid that induces a marked γδT-cell expansion and also renders patient leukemia blasts more susceptible to the lytic activity of γδT lymphocytes [14, 15]. Our present data suggest an important approach (i.e., removal of PNM-MDSC from the graft) to rescue NK-cell function for further improving the efficacy of αβT cell- and B cell-depleted haplo-HSCT. This additional graft manipulation step could have a further positive effect on the GvL activity considering that leukemia relapses still represent the main cause of mortality (>20%) [3]. In addition, it could ensure a more efficient viral protection, both these effects translating into a better patient’s clinical outcome.

## Supplementary information

Supplemental Material
