# Supplementary material for: PMN-MDSC are a new target to rescue graft-versus-leukemia activity of NK cells in haplo-HSC transplantation
Source: Leukemia. 2019 Oct 4;34(3):932–7. doi: 10.1038/s41375-019-0585-7 (PMC7214239; doi:10.1038/s41375-019-0585-7)
Supplement: Supplementary file 1 — Supplemental Material [file 41375_2019_585_MOESM1_ESM.docx]

**Supplementary Material**

**Supplementary materials and methods**

**G-CSF mobilized donors, samples, and Ethical Statements.**

Seventy G-CSF-mobilized donors were enrolled at the Onco-haematology Department of Bambino Gesù Children’s Hospital, Rome, Italy.

Details on G-CSF-mobilized donors are: 47% male, median age =40 years and 53% female, median age =37 years. Donors received G-CSF subcutaneously (10-12 µg/Kg/day) for five days before undergoing leuko-apheresis. In 8 poor mobilizing donors (circulating CD34^+^ cell count< 0.04*10^9^/L), a single dose of Plerixafor (240 µg/Kg) was added to the mobilization regimen. Peripheral blood mononuclear cells (PBMC) were obtained after density gradient centrifugation (Ficoll-Lympholyte, Cederlane). For selected functional assays, we also used leukemia blasts from patients collected at time of diagnosis and cryopreserved until their use. Both G-CSF-mobilized donors and HD gave their informed consent to participation in this study, which was approved by the Bambino Gesù Children’s Hospital (Rome, Italy) ethics committees and was conducted in accordance with the tenets of the Declaration of Helsinki.

**Cell isolation and co-culture experiments and cell lines**

After PBMC separation, CD34^+^ and PMN-MDSC cells were isolated by CD34^+^ microbeads kit ultrapure or by CD66b microbeads kit, respectively (Miltenyi Biotec) following manufacture instruction or by cell sorting (Moflo, Beckman Coulter) (purity>98%, data not shown). Pure CD34^+^ cells were cultured with RPMI 1640 (Euroclone) containing 10% AB serum (Euroclone), 100 U/ml Pennicilin (Euroclone), 0,1 mg/ml Streptomycin (Euroclone), 2mM L-glutamine (Euroclone), and cytokines including fms like tyrosine kinase 3 ligand (Flt3-L, 10ng/ml), stem cell factor (SCF), IL7, IL15 (20ng/ml) from Miltenyi and Peprotech, either in the absence or in the presence of autologous PMN-MDSC. Their differentiation potential was assessed at different time points as described in the manuscript. For intracellular cytokine detection, at the end of the culture (40 d), cells were stimulated overnight with phorbol-myristate 13-acetate (PMA 25ng/ml, Sigma Aldrich), Ionomycine (Iono, 1µg/ml, Sigma Aldrich) and IL-23 (50 ng/ml, Miltenyi Biotec) in the presence of GolgiStop and GolgiPlug (BD). NK cells were isolated from apheresis or from PB of healthy donors using NK isolation kit II (Miltenyi Biotec) or RosetteSep (StemCell technologies) (purity >95%, data not shown). Freshly isolated NK cells were immediately used or were cultured with NK medium (Miltenyi Biotec) supplemented with IL-2 (100 U/ml, Proleukin) for 10-15 days and used in the experiments as “IL-2-cultured NK cells”. Co-culture experiments were performed using NK cells cultured either alone or in combination with autologous or allogeneic PMN-MDSC under cell-to-cell contact or in transwell (Tw) conditions. Co-culture experiments were performed either in the absence or in the presence of 1-Methyl-D-Tryptophane (1-MT indolamine-2,3-Dioxygenase inhibitor, 0,25mM Sigma Aldrich) and/or N-[2-(Cyclohexyloxy)-4-nitrophenyl]methanesulfonamide (NS398, PGE2 inhibitor, 5µM Sigma Aldrich). After 48 hours PMN-MDSC were removed from co-culture using CD66b microbeads kit and the resulting NK cells used to perform phenotypical and functional assays.

**Antibodies and Flow cytometry**

For the evaluation of surface antigen expression the following monoclonal antibodies (mAbs) were used: anti-CD3-ECD, anti-CD19-ECD, anti-CD56-ECD and PC7, anti-CD11b-PC7, anti-CD33-PE, anti-HLA-DR-PerCP, anti-CD14-ECD and eFluor450, anti-CD45-APC-H7 and AlexaFluor700, anti-NKp30-PE, anti-NKp44-PC5.5, anti-NKp46-PE (Beckman Coulter), -eFluor450 (eBiosciences), anti-DNAM1-PE-Cy7 (Biolegend), anti-NKG2D-PE-CF594 (Biolegend), anti-CD16-BV510 (Biolegend), anti-CD94-FITC (Biolegend), anti-NKG2A-PE, -PC7, -APC (Beckman Coulter), anti-CD161-PE, anti-CD66b-APC, anti-CD107a-APC, anti-CD63-APC-Vio770 (Miltenyi Biotec). For intracellular and intranuclear evaluation the following mAbs were used: anti-IFN-γ PE (Miltenyi Biotec), anti-TNF-α-eFluor450 (eBiosciences), anti-IL-8-PE (R&D), anti-IL-22-PerCP-eF710, -PE (eBiosciences), anti-Granzyme B-BV421 (BD), anti-Perforin-FITC (Ancell), anti-CD3ζ-PE (Beckman Coulter), anti-DAP12-PE (R&D), anti-RORγt-PE and -APC, anti-EOMES-eFluor660 (eBiosciences). To detect transcription factors expression, cells were suspended in 5% BSA buffer, stained for surface markers, subsequently fixed with Transcription Factor Staining Buffer Set, fixed and permeabilized using FoxP3 stain buffer KIT (Miltenyi Biotec) and stained with specific mAbs. After staining procedures cells were acquired at Cytoflex S and LX (Beckman Coulter) and analyzed with Cytexpert software (v2.2, Beckman Coulter), and FlowJo 10 (TreeStar).

**Functional Assay**

To assess the degranulation, NK cells previously cultured either alone or with PMN-MDSC were incubated for 4 hours with NALM-18 cell lines or with lymphoid or myeloid patient leukemia blasts at 1:1 Effector/Target (E/T) ratio in the presence of Monensin (BD, GolgiStop) and CD107a. To detect intra-cytoplasmic cytokines and the expression of polypeptides involved in the signal transduction, cells were stained for surface markers, fixed and permeabilized with Fixation and Permeabilization Kit (BD Biosciences), and incubated with specific intracellular mAbs.

Cell cytotoxicity assays were performed using NALM-18 cell line or patients-derived leukemia blasts as target cells. Fresh NK- or IL-2 activated NK-cells previously co-cultured or not (48 hours) with PMN-MDSC were used as effector cells at different E/T ratios. In order to distinguish effector cells from target cells, NALM-18 cell line or patients-derived leukemic blasts were stained with a cell tracker following manufacture instructions (Life Technologies). Propidium iodide (PI, Sigma-Aldrich) was added at the end of the experiment (4 hours) in order to identify the percentage of target cell lysis.

**Exosome isolation and analysis**

PMN-MDSC cells were plated at 4x10^6^ cells/ml in RPMI 1640 (Euroclone) supplemented with 10% exosome-depleted Fetal Bovine Serum (FBS, Euroclone). After 48 hours, conditioned medium was collected and centrifuged at 300g for 5 min. Then, supernatant were centrifugated at 2000 *g* for 15 min, passed through a 0,22 µm filter. Exosome were pelleted by high-speed centrifugation (100’000 *g* for 2h) (Optima X Optima XPN, Beckman) and then washed with Phosphate Buffer Saline (PBS, Euroclone) for 1 hour and finally re-suspended in PBS. Exosome samples were stored at -80°C until use. Exosome protein concentration was indirectly quantified by Bradford Assay.

For the functional assays of exosomes, 5 µg of PMN-MDSC-exosomes were incubated with 0.5x10^6^ IL-2 activated NK cells. After 48 hours, the cytolytic activity (% of PI^+^ target cell killing) of exo-conditioned NK cells were assessed against NALM-18 target cells at different E/T ratios.

**Western Blot and qRT-PCR analysis**

For the Western Blot analysis, PMN-MDSC and exosome-derived PMN-MDSC were lysed in RIPA lysis buffer. Following 30 min incubation in ice, samples were centrifuged at 20000g for 20 min and supernatants collected. After Bradford assay, cell and exosome lysates were diluted in LDS-sample buffer (Thermofisher Scientific) at concentration of 10 µg under reducing or not reducing conditions. Thus, samples were run in SDS-page separation gel and transferred on nitrocellulose filters that were subsequently incubated with anti-CD63, (Santa Cruz Biotechnology), anti-Calnexin (Cell Signaling), TSG-101 (Abcam), IDO1 (Biolegend) and IDO2 (ProteinTech). Then, filters were incubated with HRP-conjugated secondary anti-mouse (Santa Cruz Biotechnology) or rabbit antibody (Cell Signaling) for 1h and signals detected with ECL method (Thermo Fisher Scientific) by Uvitec (Cleaver Scientific).

Total RNA was extracted from purified PMN-MDSC, PB-NK cells and PB-monocytes using miRNeasy mini kit (Qiagen). Random hexamer-primed cDNA was prepared using SuperScript IV first strand Synthesis System following manufacturer's instructions ((Thermo Fisher Scientific, Wilmington, DE, U.S.A.). Real time PCR were carried out in triplicate with PowerUp™ Sybr® Green reagent (Applied Biosystems, Foster City, CA, U.S.A). Beta-actin (ACTB) was used as endogenous control. The following primers were used: IDO1 (FW: 5’-GGGAAGCTTATGACGCCTGT-3’; RV: 5’-CTGGCTTGCAGGAATCAGGA-3’), IDO2 (FW: 5’-GCGGAGCTATCACATCACCA-3’; RV: 5’-GGAGATGGTTTGGCTTCCCA-3’) ACTB (FW: 5’-ACCGCGAGAAGATGACCCAGA-3’; RV: 5’-GGATAGCACAGCCTGGATAGCAA-3’).Real time PCR were carried out on a QuantStudio 6 Flex instrument (Applied Biosystems, Foster City, CA, U.S.A). Expression values were calculated by ΔΔCt method for comparing relative fold expression differences using QuantStudio Real-Time PCR system software v. 1.3.

**Confocal microscopy analysis**

For confocal microscopy analysis, NK cells were labeled with PE-conjugated anti-CD56 (Beckman Coulter) antibody and DAPI staining (Vectorlabs) as previously described[^46^](#_ENREF_46) while PMN-MDSC-exosomes were labeled with PKH-67 dye (Sigma-Aldrich) according to previous study[^47^](#_ENREF_47). Then, NK cells were incubated with PKH^+^ PMN-MDSC-exosomes for 48 hours in order to evaluate exosome uptake. Images were acquired by a Leica TCS-SP8X Laser-scanning confocal microscope (Leica Microsystem) equipped with tunable white light laser (WLL) source, 405nm diode laser, 3 Internal Spectral Detector Channels (PMT) and 2 Internal Spectral Detector Channels (HyD) GaAsP. Data were analyzed using LAS X3D software (Leica Microsystem) and images were processed Adobe Photoshop CS4 software (Adobe Systems Inc.).

**Statistical Analysis**

Statistical analysis were performed with GraphPad Prism software 6.0. In Figure 1B-C, 2A, B and Q we used nonparametric Mann–Whitney test. Figure 1E, show one-way ANOVA post test for linear trend. Figure 2E shows the two-way ANOVA test. Figure 2, H, I, J show the nonparametric Wilcoxon tests. A p value ≤ 0.05 was considered statistically significant. *p ≤ 0 .05; **p ≤ 0 .01; *** p ≤ 0.001; **** p ≤ 0.0001; ns= not significant. Where not indicated, the data were not statistically significant.

**Supplementary Figure**

**
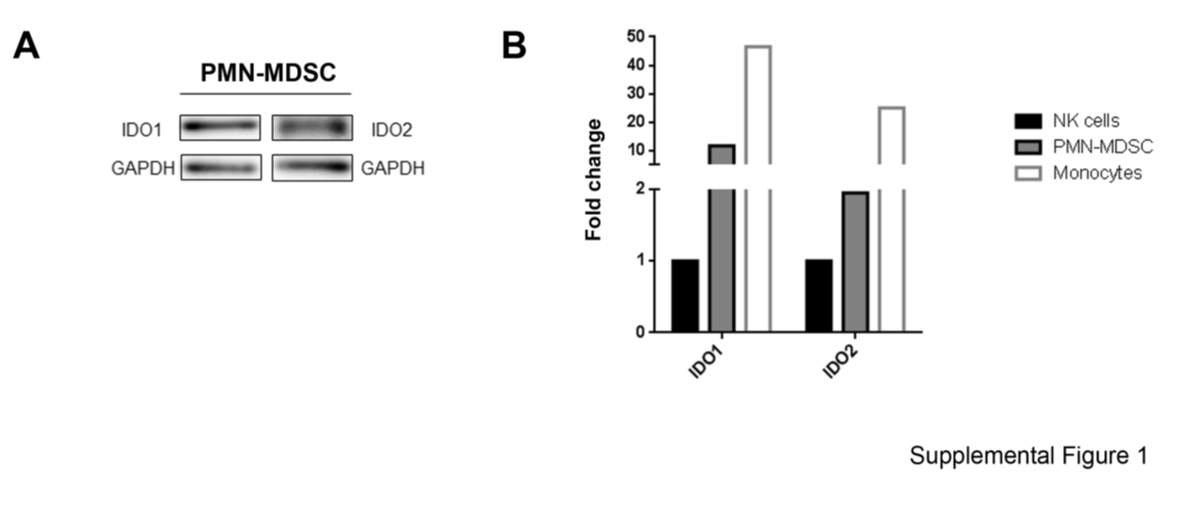
**

**Supplementary Figure 1. Presence of IDO in PMN-MDSC.** (A) Western blot analysis of IDO1 and IDO2 expression on purified PMN-MDSC. GAPDH was used as control. (B) Evaluation by Real-Time PCR of IDO1 and IDO2 mRNA in purified PMN-MDSC and, as control, freshly isolated monocytes and NK cells. For each group of cells, we calculates sample relative expression based on the expression level detected on NK cell arbitrarily normalized to 1. In all samples, we normalized gene expression levels to β-Actin mRNA.
